# Migraine pain location in adult patients from eastern India

**DOI:** 10.4103/0972-2327.41876

**Published:** 2008

**Authors:** Ambar Chakravarty, Angshuman Mukherjee, Debasish Roy

**Affiliations:** Department of Neurology, Vivekananda Institute of Medical Sciences, Kolkata, India

**Keywords:** Adult migraine, migraine pain, pain location

## Abstract

**Background:**

Sparse literature documenting the location of pain at the onset of migraine attacks and during established headaches is available.

**Objectives:**

A prospective study (2003–05) on 800 adult migraine patients (International Classifications of Headache Disorders (ICHD), 2:1.1, 1.2.1 and 1.6.1) was conducted to document (a) sites of onset of pain and (b) location of pain during established attacks (in >50% occasions) through semistructured interviews.

**Results: Demography:**

N = 800; M:F = 144:656 (1:4.56); age, 16–42 years (mean, 26 years); duration of migraine, 1–18 years (mean, 6.8 years). 87% of the subjects were ethnic Bengalis from the eastern Indian state of West Bengal, Calcutta being the capital city.

**Migraine types:**

(on the basis of >50% headache spells): N = 800; 1.1:668 (83.5%); 1.2.1:18 (2.25%); 1.6.1:114 (14.25%).

**Location of pain at onset:**

Unilateral onset was present in 41.38% of the patients; of these, 53.17% had eye pain; 8.16%, frontal pain and 38.67%, temporal pain. In 32.25% of the patients, bilateral/central location of pain, mostly bitemporal or at vertex was noted. Cervico-occipital pain onset was noted in 26.43% patients (predominantly occipital, 14.68%; predominantly cervical, 11.75%).

**Location of established headaches:**

In 47.4% of the patients, with unilateral ocular or temporal onset, pain remained at the same site. Pain became hemicranial in 32.9%. In most patients, unilateral frontal onset pain (55.5%) became bilateral or holocranial. Most bilateral ocular (69.4%) and temporal onset (69.7%) pains remained at the same location. However, most bifrontal (55.6%) and vertex onset (56.9%) pains subsequently became holocranial. Most occipital pains at onset became holocranial (45.3%), but cervical pains subsequently became either hemicranial (38.3%) or holocranial (36.2%).

**Conclusions:**

This study documents location of pain at the onset and during established headaches in migraine patients largely from a specific ethnic group. Migraine with aura appears to be rare among ethnic Bengalis in eastern India. More than half had onset pain bilaterally/centrally and in the cervico-occipital regions. Only 40.5% experienced only unilateral pain. Cervico-occipital migraine pain appears to be common in ethnic Bengalis.

## Introduction

Hemicranial location of pain as a cardinal feature of acute migraine attack has been recognized since ancient times, and the origin of the term ‘migraine’ had been linked to this observation. This notion has perpetuated through centuries, and a unilateral location of pain has been included in the diagnostic criteria of migraine (both with and without a typical aura) by the International Headache Society (IHS) according to the International Classifications of Headache Disorders (ICHD-1 and ICHD-2) conducted in 1988 and 2004, respectively.[1,2] Critical review of published reports however reveal that location of migraine pain at the onset, its subsequent spread and the final location during established headaches have not yet been studied in adequate detail involving large number of subjects.[3,4] Experience suggests that even in an individual subject, the location of pain may not remain exactly the same during all attacks. Moreover, it is possible that ethnic variation in the locations may exist. Migraine pain has been reported to be hemicranial in approximately 60% patients.[4] Sjaastad *et al.*, noted localization of initial pain in both ‘classical’ and ‘common’ migraine attacks in the forehead and temporal regions (although ‘ocular’ onset pain has been mentioned with regard to migraine without aura).[5,6] These authors have independently published that unilaterally with side shift of pain was present in 75% of subjects with ‘common’ migraine.[7] These observations have been made in relatively smaller number of subjects and may not necessarily be applicable to all ethnic groups and during all or at least most of the migraine attacks in the lifetime of an individual. Probably the only detailed report on the location of migraine pain and assessment of the impact of gender, types of migraine, aura and other headache features, has recently been prepared by Kelman.[8] This study did not specifically address the location of pain at the onset or the temporal evolution of the location. The present study was designed and had been in progress since long before Kelman's publication and attempted to document the site of onset of migraine pain and its subsequent location during established headache in a large cohort of patients mostly belonging to a specific ethnic group visiting the clinic.

## Materials and Methods

The present study was carried out between September 2003 and December 2005 at the neurology outpatients department (tertiary care center) of a large general hospital (550 beds) in Calcutta in eastern India, which is largely dominated by ethnic Bengali population. We assessed a total of 1172 adult patients (>15 years of age) by using a semi-structured questionnaire; these patients were diagnosed with episodic migraine attacks and experienced at least 2 attacks of acute migraine every month during the preceding 6 months of enrollment and were not on any form of prophylactic medication for the same period. Diagnoses of migraine and migraine subtypes were made according to the ICHD-2 criteria available on the web in 2003 and later published in February 2004. The assessment was made through several stages.

At the first visit, each patient was evaluated by one of the three participating consultants (authors) to confirm the diagnosis of migraine, identification of subtype and arranging relevant investigations to exclude secondary headaches as and when required (imaging performed in 162 patients and showed positive result in only two patients).

During the second visit, one week (on average) later, each patient was evaluated using a semi-structured questionnaire by a trainee physician specifically assessing the site of location of the onset pain and its subsequent location during established attacks in the majority (>50%) of headache spells in the preceding six months. The patients were requested to place their hands at the sites of onset pain, indicate with their hands the subsequent spread of the pain (if possible) and finally the location of pain during the established phase of the headache. Descriptions of the terminologies associated with anatomical sites are presented at the end of this section.

During a third visit, usually a month later, each patient was asked the same question regarding the location of pain by one of the authors (consultants) and the responses compared to those given in the interview by the trainees. In subjects in whom some discrepancies were noted between the two responses, the patients were offered the first responses and given the choice to be really specific. At this stage, patients (n = 102) providing grossly discrepant and vague responses (not really of localizing value) were excluded from the study. Moreover, patients who failed to turn up for the second or third stage of interview (even after reminder) were excluded at this stage.

Finally, all response sheets were carefully evaluated by the corresponding author and only the first 800 subjects providing consistent (or near consistent) responses to questions associated with pain localization both at the onset and during the established phase of headache, were included for analysis.

The study includes only patients with episodic migraine and not those presenting with chronic migraine (ICHD-2 criteria). Pain in most of these patients was found to be rather poorly localized at the onset and during the ongoing headache phase.

Description of terms used for anatomical sites for the location of pain:

1.Ocular:Eye/Orbit2.Frontal:Unilateral – one side of forehead Bifrontal – whole of forehead3.Temporal:Unilateral/Bilateral temporal region4.Hemicranial:Unilateral temporal and parietal regions; may involve ipsilateral frontal/orbital regions and occasionally ipsilateral occipital area5.Vertex/Central:Center of the cranium6.Occipital:Back of head above hairline – generally both sided7.Cervical:Back of neck below hairline – generally both sided8.Holocranial:Whole forehead, temporal and parietal as well as central cranial area and occipital region

It may be noted that, while noting the location of pain, especially at the onset, the area mostly affected was taken into account. For example, pain involving one side of forehead but extending slightly into the temporal region, had been designated as frontal onset and similarly vice versa. Indeed, this may be somewhat artificial, but this was necessary for simplifying the final analysis a little but maintaining its significance.

## Results

The demographic and ethnic characteristics of the subjects are described in Table 1. Most of the subjects had migraine without aura, while only less than 3% subjects had migraine with aura [Table 1]. The location of headache is described in Table 2. The relationship between the site of headache and the onset of headache is presented in Table 3.

**Table 1:** Migraine in adults: Demography and clinical types (ICHD-2) [N = 800]

M:F	:	144:656 (1:4.56)
Age	:	16–42 years (mean, 26 years)
Duration of migraine	:	1–18 years (mean, 6.8 years)
Ethnicity	:	Ethnic Bengalis: 696 (87) Others: 104 (13)
Migraine without aura (1.1)	:	668 (83.5)
Migraine with typical aura (1.2.1)	:	18 (2.25)
Probable migraine (1.6.1)	:	114 (14.25)

Figures in parentheses indicate percentages

**Table 2:** Location of pain at migraine onset (in >50% occasions) [N = 800]

A.	Unilateral onset	:	331 (41.38)
	1. Ocular	:	176 (53.17)
	2. Frontal	:	27 (8.16)
	3. Temporal	:	128 (38.67)
	Laterality at onset (n = 331)	
	1. Always right	:	6 (18.73)
	2. Always left	:	43 (12.99)
	3. >50% right	:	132 (39.88)
	4.>50% left	:	94 (28.4)
B.	Bilateral/Central onset	:	258 (32.25)
	1. Bilateral ocular	:	36 (13.96%)
	2. Bifrontal	:	18 (6.97%)
	3. Bitemporal	:	132 (51.16)
	4. Vertex	:	72 (27.91)
C.	Cervico-occipital onset	:	211 (26.38)
	1. Predominantly occipital	:	117 (54.45)
	2. Predominantly cervical	:	94 (45.55)

Figures in parentheses indicate percentages

**Table 3:** Location of pain during established headache phase with regard to the location at onset [N = 800]

Onset site	Location during established headache		
A. Unilateral (n = 331)			
1. Ocular (n = 176)	a. Remained same	:	81 (46)
	b. Hemicranial	:	65 (36.9)
	c. Bilateral/Holocranial	:	30 (17)
2. Frontal (n = 27)	a. Remained same	:	8 (29.6)
	b. Hemicranial	:	4 (14.8)
	c. Bilateral/Holocranial	:	15 (55.5)
3. Temporal (n = 128)	a. Remained same	:	68 (53.1)
	b. Hemicranial	:	40 (31.3)
	c. Bilateral/Holocranial	:	20 (15.6)
B. Bilateral/Central (n = 258)			
1. Bilateral ocular (n = 36)	a. Remained same	:	25 (69.4)
	b. Holocranial	:	11 (30.6)
2. Bifrontal (n = 18)	a. Remained same	:	8 (44.4)
	b. Holocranial	:	10 (55.6)
3. Bitemporal (n = 132)	a. Remained same	:	92 (69.7)
	b. Holocranial	:	40 (30.3)
4. Vertex (n = 72)	a. Remained same	:	22 ((30.6)
	b. Hemicranial	:	9 (12.5)
	c. Holocranial	:	41 (56.9)
C. Predominantly occipital (n = 117)	a. Remained occipital	:	23 (19.7)
	b. Cervico-occipital	:	28 (23.9)
	c. Hemicranial	:	13 (11.1)
	d. Holocranial	:	53 (45.3)
D. Predominantly cervical (n = 94)	a. Remained cervical	:	9 (9.6)
	b. Cervico-occipital	:	15 (15.6)
	c. Hemicranial	:	36 (38.3)
	d. Holocranial	:	34 (36.2)

Figures in parentheses indicate percentages

Our main observations were the following: migraine with aura is uncommon in Indian patients, especially amongst ethnic Bengalis in eastern India (18/800, i.e., 2.25% [Table 1]). Approximately 41.38% of subjects had a unilateral onset of migraine headache of which slightly over half (53.17%) had an ocular location of pain at the onset and slightly over one-third (38.67%) had location of pain at the onset in the temporal region. In approximately one-third of subjects with unilateral onset, pain had been side-locked. Bilateral/central (vertex) location of pain at the onset was experienced by 32.25% of the subjects. A majority (51.16%) of them had bitemporal pulsating pain in the beginning followed by 27.91% experiencing onset pain at the vertex region. There were 26.43% of subjects who had a cervico-occipital location of pain at the onset, of which 14.63% had predominantly occipital region onset and 11.75% had predominantly cervical region onset of pain. As in many cases, it was difficult to clearly distinguish between the cervical or occipital location of onset pain; these two areas were grouped together and divided into predominantly cervical and occipital ones. In 47.4% of the subjects with unilateral ocular, frontal and temporal location of pain at the onset, pain remained at the same site during established headaches. Approximately one-third of subjects with the location of pain at the onset in unilateral ocular, frontal and temporal regions subsequently developed ipsilateral hemicranial pain during established spells of migraine headaches. Only 19.6% of the subjects with unilateral location of pain at the onset developed bilateral or holocranial headaches during the established phase of the migraine attack. In 56.98% of the subjects with bilateral or vertex location of pain at the onset (n = 258), pain remained at the same site during established migraine attacks. Approximately 70% of the subjects with bitemporal onset of throbbing pain, continued to experience pain at the same site but in approximately 30% pain subsequently became holocranial. Over half (56.9%) of the subjects having onset pain at the vertex region subsequently experienced holocranial headaches. There were 41.23% of subjects with cervico-occipital location of pain at the onset subsequently experienced holocranial headaches. Over one-third (38.38%) of the subjects having predominantly cervical location of pain at the onset but only 11.1% of those having predominantly occipital location of pain at the onset subsequently experienced hemicranial headaches. Among all subjects, although during established migraine attacks, 40.5% of subjects experienced only unilateral pain, only 20.88% had only hemicranial headaches.

## Discussion

The present study was carried out among adult migrain patients attending the neurology outpatient department of a general teaching institution and comprised largely (87%) of subjects belonging to a specific ethnic origin (Bengalis residing in the eastern Indian state of West Bengal, the capital city being Calcutta). It appears that apparently no previous study on clinical aspects of migraine has taken into account the ethnicity of the subjects.

Apart from Kelman's recent report,[8] no other study in the past has studied in detail of the location of migraine pain as had been conducted in the present study. However, there are significant differences between the observations made in Kelman's study[8] and the present one. Study population consists largely of one specific ethnic group, cases with chronic migraine had not been included, and no patient was on any form of prophylactic drug treatment.

There are two important observations that deserve special attention. In Indian patients with migraine (specially in ethnic Bengalis from eastern India), only unilateral headache occurred in only 40.5% patients and true hemicranial headache (occulo-fronto-temporoparietal or fronto-temporoparietal) occurred in only one-fifth of all the subjects on most (>50%) occasions of migraine attacks[9]. These figures are in sharp contrast to the overall experience in the West,[10,11] including that of Kelman's report[8] (hemicranial headache in 71.2% of the subjects with episodic migraine and in over 60% of those with chronic migraine). In this context, the inclusion of unilaterality of migraine headaches in the diagnostic criteria (ICHD-2) probably needs careful reconsideration. In approximately 31% of subjects with unilateral onset of migraine, pain was side-locked. This figure is slightly higher than those reported previously (17–20.8%).[12–14] However, these figures were based on smaller number of patients. Side-locked pain onset was not assessed in Kelman's study.[8]

The location of migraine pain at the onset in the cervico-occipital region (noted in over a quarter of subjects in the present series) is noteworthy, especially the occurrence of predominantly cervical location in over 10% of subjects. This had not been adequately addressed to in the western literature, although Kelman did note this in a recent communication.[8] Indeed, most of the subjects in the present study with cervico-occipital location of pain at the onset, subsequently experienced either holocranial or hemicranial headaches. However, less than 10% of all subjects with migraine only experienced cervico-occipital pain on most (>50%) occurrences of established migraine attacks.[9] Overlapping clinical aspects between common migraine and cervicogenic headaches had been described previously by Sjaastad *et al*,[7] but the concept of cervicogenic headache appears very different now (in ICHD-2) than it was over a decade ago. The ethnic variation in the disease phenomenology needs recording and may provide insights into pathophysiology as discussed below.

Most migraine pains noted in the present series appeared to have an ‘anterior’ location, as noted in nearly all studies conducted in the West. This would be consistent with the generally agreed pathophysiology of migraine with trigeminovascular activation involving the first division of the trigeminal nerve. Kelman[8] suggested that cervico-occipital localization could be due to the phenomenon of ‘convergence’ of sensory input ‘rather similar to the left medial arm pain in myocardial ischemia’. We would prefer to offer a more easily understandable explanation. The sensory nucleus of the trigeminal nerve, the central processing unit of all head and facial pain, extends up to the C2 segment of the upper cervical cord. The C2 segment, through the corresponding motor and sensory roots innervates most structures in the cervico-occipital region, including the walls of the blood vessels in the same region. A cervico-trigemino-vascular reflex may well be in operation.

It is expected that similar clinical studies associated with the location of migraine pain would be carried out in different geographic regions and in different ethnic groups to highlight global variability in the phenotypic expression of this common disorder.
